# Association between Immune Related Adverse Events and Outcome in Patients with Metastatic Renal Cell Carcinoma Treated with Immune Checkpoint Inhibitors

**DOI:** 10.3390/cancers13040860

**Published:** 2021-02-18

**Authors:** Agnese Paderi, Roberta Giorgione, Elisa Giommoni, Marinella Micol Mela, Virginia Rossi, Laura Doni, Andrea Minervini, Marco Carini, Serena Pillozzi, Lorenzo Antonuzzo

**Affiliations:** 1 Clinical Oncology Unit, Careggi University Hospital, Largo Brambilla 3, 50134 Florence, Italy; paderi.agnese@gmail.com (A.P.); roberta.giorgione@unifi.it (R.G.); elisa.giommoni@unifi.it (E.G.); melam@aou-careggi.toscana.it (M.M.M.); virginiarossi89@gmail.com (V.R.); donila@aou-careggi.toscana.it (L.D.); serena.pillozzi@unifi.it (S.P.); 2 Urology Unit, Careggi University Hospital, Largo Brambilla 3, 50134 Florence, Italy; andrea.minervini@unifi.it (A.M.); marco.carini@unifi.it (M.C.); 3 Department of Experimental and Clinical Medicine, University of Florence, Largo Brambilla 3, 50134 Florence, Italy

**Keywords:** renal cell carcinoma, immune checkpoint inhibitors, immune related adverse events (irAEs), thyroid, cutaneous, biomarker

## Abstract

**Simple Summary:**

Patients treated with immune-checkpoint inhibitors often experience a wide range of peculiar adverse events, called immune-related adverse events (irAEs). Lately, it has been described that the presence of irAEs may be associated with better clinical response to immunotherapy. The aim of our retrospective study was to observe the onset of the most common side effects and to evaluate their potential prognostic impact in a cohort of metastatic renal cell cancer patients treated with immunotherapy. We confirmed a correlation between irAEs and progression free survival in patients with cutaneous and thyroid adverse reactions as well as in patients that experienced two or more irAEs. Thus, the development of irAEs could act as a clinical marker of efficacy in metastatic renal cell patients treated with immunotherapy.

**Abstract:**

Background: It has been reported that the occurrence of immune-related adverse events (irAEs) in oncological patients treated with immune-checkpoint inhibitors (ICIs) may be associated with favorable clinical outcome. We reported the clinical correlation between irAEs and the efficacy of ICIs in a real-world cohort of metastatic renal cell cancer (mRCC) patients. Methods: We retrospectively evaluated 43 patients with mRCC who were treated with nivolumab or with nivolumab plus ipilimumab. We considered seven specific classes of irAEs including pulmonary, hepatic, gastrointestinal, cutaneous, endocrine, rheumatological, and renal manifestations. We assessed progression-free survival (PFS) of specific irAEs classes compared to the no-irAEs group. Results: Twenty-nine out of 43 patients (67.4%) experienced a total of 49 irAEs registered. The most frequent irAE was thyroid dysfunction (*n* = 14). The median PFS after the beginning of therapy was significantly longer in patients with thyroid dysfunction and cutaneous reactions. In multivariate analysis, thyroid dysfunction was an independent factor for favorable outcome [HR: 0.29 (95% CI 0.11–0.77) *p* = 0.013]. Moreover, experiencing ≥2 irAEs in the same patient correlated in multivariate analysis with better outcome compared with none/one irAE [HR: 0.33 (95% CI 0.13–0.84) *p* = 0.020]. Conclusions: This retrospective study suggests an association between specific irAES (thyroid dysfunction and skin reaction) and efficacy of ICIs in metastatic RCC. Notably, multiple irAEs in a single patient were associated with better tumor response.

## 1. Introduction

The treatment scenario of metastatic renal cell cancer (mRCC) has undergone a complete change in the last few years. Therapeutic options have progressed from non-specific immunotherapy with cytokines to targeted therapy with the development of tyrosine kinase inhibitors (TKI), and more recently to the novel immune checkpoint inhibitors (ICIs) such as anti-programmed death receptor 1 (PD-1), anti-programmed death receptor ligand 1 (PD-L1), and anti-cytotoxic T lymphocytes antigen 4 (CTLA-4) [1]. The CheckMate 025 was the first trial showing the efficacy of nivolumab, a human IgG4 anti-PD-1 antibody, in mRCC patients [2]. Later, the CheckMate 214 trial investigated the combination of nivolumab with another ICI, ipilimumab (anti-CTLA-4), and confirmed the benefit of the association, especially in patients with intermediate and poor-risk disease according to the IMDC (International Metastatic RCC Database Consortium) risk score [3]. More recently, the combined use of an ICI on top of a multitargeted receptor TKI evidenced a survival benefit and is therefore now another therapeutic option for patients with mRCC [4,5,6]. The opportunities to use ICIs in the future will, most likely, tremendously increase.

With the number of mRCC patients treated with immunotherapy rising year by year, clinicians have found themselves managing a new spectrum of adverse events (AEs) that are specific to this new class of therapeutic agents [7]. As expected, by stimulating the immune system to target malignancies, ICIs have also induced a wide range of immunologic AE (irAEs). The most common reported irAEs involve skin, gastrointestinal tract, endocrine glands, lung, and liver [8]. Little is known about the cellular and molecular mechanisms underlying most irAEs. However, emerging evidence from clinical trials and real-world studies indicate that irAE type and severity depend on the therapeutic target (i.e., CTLA-4 vs. PD-1/PD-L1), tumor type, and patient-intrinsic factors [9,10]. Most irAEs are mild and reversible if detected early and properly managed, but there is a noticeable proportion of patients who have experienced (grade ≥ 3) irAEs (31% of patients treated with CTLA-4 vs. 10% treated with PD-1) [11]. It has been reported that the incidence of irAEs is higher in ICI combination therapy than in monotherapy [12]. It must be noted that most clinical trials excluded cancer patients with underlying autoimmune disease or chronic infection. Therefore, irAEs in real-world clinical practice are expected to increase further.

However, despite the improved outcome of cancer treatment by ICIs, efficacy still remains limited. Many studies have searched for biomarkers predictive of response to immunotherapy. Some of these are already used in clinical practice including the PD-L1 tumor prediction score (TPS) and clinical prediction score (CPS) [13,14]. Other markers currently under evaluation include tumor mutational burden (TMB) [15], gene expression scores (GEP) [16], and tumor infiltrating lymphocytes (TILs) [17]. Other studies have suggested that peripheral blood markers could help predict the treatment response, but evidence is still scant [18,19,20]. In the context of this research irAEs have been proposed as potential clinical markers to predict response to ICI. This relationship, although documented in studies regarding non-small cell lung cancer (NSCLC) and melanoma [21,22,23,24,25,26] has been less studied in RCC [27,28,29]. Moreover, current data on the impact of specific types of irAEs on outcomes are not entirely consistent.

Our study represents a real-life observation concerning the onset and management of the most common side effects and the prognostic impact of irAEs in a cohort of mRCC real-world patients treated with nivolumab or nivolumab combined with ipilimumab at our Medical Oncology Unit, AOU Careggi (Firenze, Italy).

## 2. Materials and Methods

We retrospectively reviewed data from 43 patients treated at the Medical Oncology Unit, Careggi Hospital (Firenze, Italy) from March 2016 to March 2020.

Patient eligibility included age >18, histologically confirmed RCC with metastatic disease, and treatment with an immunotherapy agent. Patients were treated with nivolumab in monotherapy (3 mg/kg every two weeks or flat dose of 240 mg/every two weeks or 480 mg/every four weeks) or with the association of nivolumab 3 mg/kg and ipilimumab 1 mg/kg every three weeks for four cycles, then in monotherapy with nivolumab. PD-L1 status was not requested. Patients received therapy until either disease progression or unacceptable toxicity presented. All patients had measurable disease and the disease progression was evaluated according to the Response Evaluation Criteria in Solid Tumors (RECIST v 1.1).

All patients evaluated had accurate clinical records of the irAEs with a description of their severity and their treatment. Toxicity was assessed according to Common Terminology Criteria for Adverse Events (CTCAE) version 4.0. IrAEs were defined as an adverse event with an immunological basis that required intensive monitoring or treatment with immunosuppressive agents or endocrine therapy. We divided irAEs into seven categories: pulmonary, hepatic, gastrointestinal, cutaneous, endocrine, rheumatological, and renal.

All data were analyzed anonymously; all patients signed an informed consent form for immunotherapy with particular specifications about the occurrence of possible adverse events. This study was conducted in accordance with the World Medical Association Declaration of Helsinki and independently reviewed and approved by the Regional Ethics Committee for Clinical Trials of the Tuscany Region (approval no.: 17332_oss). All patient data were processed anonymously and de-identified prior to analysis.

Statistical analyses. Estimates of PFS in the irAE and non-irAE groups or different irAEs subgroups were calculated using the Kaplan–Meier method, and statistical significance was analyzed using the log-rank test. A significance level of *p* < 0.05 was employed for statistical analyses. First, at the univariate analysis, and then the Cox proportional hazard model was used to calculate the hazard ratios (HRs) and appropriate 95% CIs. Subsequently, the independent effect of each parameter on PFS was investigated by a multivariate Cox regression model. Data were analyzed using the statistical software Jamovi.

## 3. Results

### 3.1. Patient Characteristics

Between March 2016 and March 2020, 43 patients with mRCC were treated with ICIs (either nivolumab or nivolumab combined with ipilimumab) at our Medical Oncology Unit, AOU-Careggi (Florence). Table 1 summarizes the patients’ clinical features.

Average age of enrolled patients at time of diagnosis was 64 years, ranging from 45 to 79 years; 81.4% (*n* = 35) male, and 18.6% (*n* = 8) female. A total of 53.4% of the patients were current or former smokers. The most frequent histology diagnosis was clear cell (83.7%), followed by papillary and chromophobe renal cancer. Most patients (*n* = 39/43, 91%) had a resected primary tumor. The most frequent site of metastases was lung (65% of patients), followed by lymph nodes (44%), bone (35%), liver (21%), and brain (7%).

Overall, 33 patients (76.7%) received nivolumab in monotherapy while 10 received (23.7%) nivolumab plus ipilimumab. Ten patients (23.2%) received ICI as the first line of therapy and 25 patients (58.1%) as second line. Overall, one patient achieved complete response (2.3%), six partial response (PR) (13.9%), 11 stable disease (SD) (25.6%), and the remaining 25 patients experienced progressive disease (PD) (58.1%).

Among patients treated with nivolumab (*n* = 33), 18 (54.5%) were non responders (defined as patients who experienced a progressive disease as best response), while among patients treated with nivolumab plus ipilimumab (*n* = 10), seven (70%) were non responders.

### 3.2. Profile of Immune-Related Adverse Event (irAEs)

Twenty-nine out of 43 patients (67.4%) experienced a total of seven different irAE categories (data are reported in Table 2). Baseline clinical features between patients with or without irAEs were not significantly different. In patients who developed irAEs, the median number of days before the onset was 59 days; 14 patients never developed irAEs until the end of the observation phase.

In total, we registered 49 irAEs: 20 (46.5%) developed endocrine-related events, nine (20.9%) developed skin reactions, seven (16.3%) developed hepatitis, five (11.6%) developed colitis (as diarrhea), four (9.3%) developed arthralgias/myalgias, three patients (9%) developed pneumonia, and one patient (2.3%) developed nephritis.

According to the CTCAE grades, irAEs registered were mainly grades 1 and 2, and were included in a subgroup defined “non-serious AE”. Within patients who developed any irAE, 19 experienced a non-serious irAE (65.5%), while 10 patients (34.5%) developed a serious irAE (grade 3 or grade 4). If we consider the total number of irAEs observed (*n* = 49), 12 irAEs were serious (24.5%), while 37 were classified as non-serious (75.5%).

Among the patients only treated in monotherapy with nivolumab (*n* = 33), three (9%) developed pneumonitis, five (15.1%) developed diarrhea, four (12.1%) developed hepatitis, eight (24.4%) developed skin reactions, four (12.1%) developed arthralgia/myalgia, and 14 (42.4%) developed endocrine-related events.

Regarding the cohort of patients treated with nivolumab in combination with ipilimumab (*n* = 10), three (30%) patients developed hepatitis, only one (10%) patient developed a skin reaction, six (60%) patients developed endocrine-related events, and one (10%) patient developed nephritis. No difference in the occurrence of AEs between mono and combination therapy could be found (*p* = 0.84).

Overall, the most frequent AEs were related to endocrine issues (46% of the patients with 19 events), with a percentage up to 40% of the total number of adverse events. The median time to first development of endocrine dysfunction was 80 days. Thyroid dysfunction was by far the most frequently encountered endocrine toxicity (*n* = 15/19), followed by hypophysitis and hyperglycemia. Some patients (*n* = 8) experienced early-stage thyrotoxicosis followed by a permanent stage of hypothyroidism.

### 3.3. Relationship between irAEs and Patient Outcome

**Figure 1:**
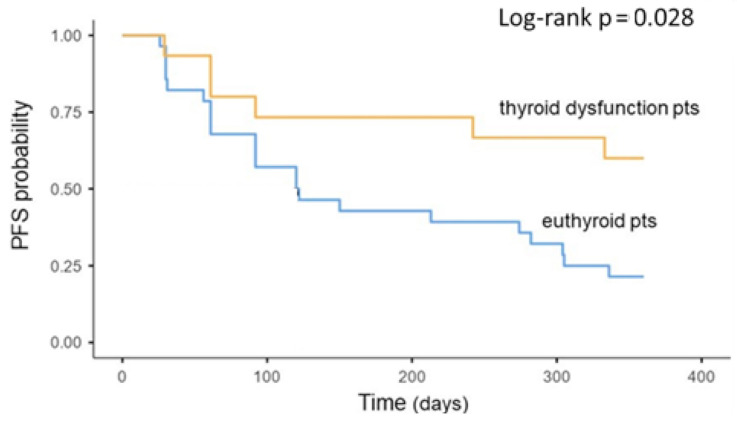
Association between thyroid dysfunction during treatment and oncologic outcomes. Kaplan–Meier plots of progression-free survival. IrAEs = immune-related adverse events; PFS = progression-free survival; pts = patients.

PFS was significantly longer in the group of patients that developed a thyroid dysfunction (*p* = 0.028); the median PFS of the euthyroid group was 121 days (IQR 92–305.) while the median PFS of the thyroid dysfunction group was not reached (Figure 1).

In multivariate analysis, the development of thyroid toxicity was an independent prognostic factor for PFS (HR: 0.34 [95% CI 0.13–0.87] *p* = 0.025) (Table 3). On the other hand, although very rare, hypophysitis was related to significant shorter PFS (*p* = 0.048). Interestingly, endocrine toxicities were significantly higher in patients who had already performed two lines of therapy before immunotherapy (*p* = 0.022).

**Figure 2:**
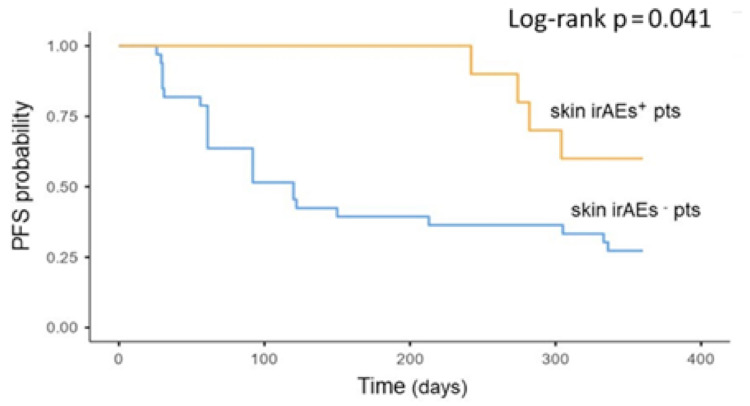
Association between skin toxicity during treatment and oncologic outcomes. Kaplan–Meier plots of progression-free survival. irAEs = immune-related adverse events; PFS = progression-free survival; pts = patients.

A significantly longer PFS was also found in patients who experienced skin irAEs (*p* = 0.41). The median PFS of the patients who did not experience skin toxicities was 120 days (IQR 61–336), while the median PFS of the skin irAE group was not reached (Figure 2).

**Figure 3:**
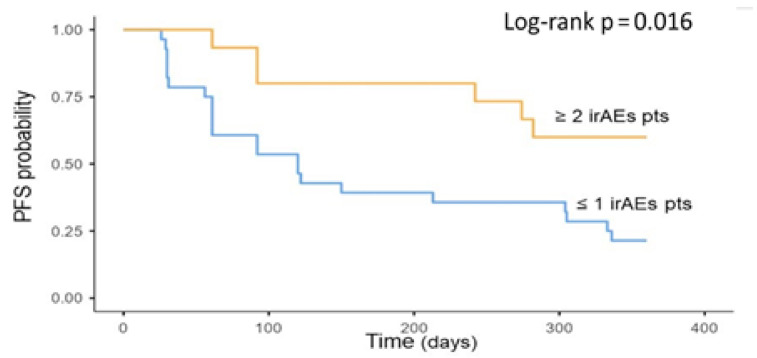
Association between multiple irAEs during treatment and oncologic outcomes. Kaplan–Meier plot of progression-free survival. irAEs = immune-related adverse events; PFS = progression-free survival; Pts = patients.

Moreover, in the irAEs group, the PFS was significantly longer in patients who experienced two or more irAEs compared to only one or no irAEs (*p* = 0.016) (Figure 3). In multivariate analysis, the development of two or more irAEs was an independent prognostic factor for PFS (HR: 0.32 [95% CI 0.13–0.79] *p* = 0.014) (Table 3). Age, sex, current or former smoking status, and pathological subtypes were not associated with PFS.

Additionally, we performed a complementary 16-week landmark analysis since patients with longer PFS could have a higher probability of developing AEs, which could lead to analysis bias. The 16-week analysis confirmed that the occurrence of irAEs was significantly associated with prolonged median PFS for patients with skin toxicity and with two or more adverse events (PFS: NR vs. 120 days, *p* = 0.005 and PFS: NR vs. 120 days, *p* = 0.029, respectively) The same analysis for patients with thyroid dysfunction demonstrated similar tendencies, but was not statistically significant (*p* = 0.160).

## 4. Discussion

Immunotherapy potentiates a patient’s immune system to fight cancer, and has become one of the standard treatments for RCC. Immune checkpoint blockade increases antitumor immunity by blocking intrinsic downregulators of immunity such as cytotoxic T-lymphocyte antigen 4 (CTLA-4) and programmed cell death 1 (PD-1) or its ligand, programmed cell death ligand 1 (PD-L1). By enhancing the activity of the immune system, immune checkpoint blockade can induce inflammatory side effects.

Within this study, we aimed to describe toxicities in a real-world cohort of mRCC patients and their potential association with treatment response.

The precise pathophysiology underlying irAEs is yet unknown, but various hypotheses have been made. Some potential mechanisms include increasing T cell activity against antigens that are present both in tumors and healthy tissue and increasing levels of preexisting autoantibodies [30]. Among other theories, the observation that gut microbiota is involved in the functions of intestinal CD4+ and CD8+ T, with anti-tumor immunological activity, is gaining strength and this could impact the efficacy of ICIs [31].

It must be noted that CTLA-4 and PD-1 inhibit the immune response in distinct ways, the first attenuating T-cell activation at a proximal step in the immune response [32] and the latter blocking T cells at later stages of the immune cascade in peripheral tissues [33]. These different functions are reflected in different toxicities. For instance, pneumonitis and thyroiditis appear to be more common with anti-PD-1 therapy, while colitis and hypophysitis seem to be more common with anti-CTLA-4 therapy [11].

Overall, our results confirmed a favorable toxicity profile in patients with mRCC as described in other real-life studies [28,34]. However, colitis was unexpectedly higher in the nivolumab group, however, the total number of cases was very low. We also found a higher frequency of AEs on the endocrine profile compared to previous studies, reaching 46% in our patients compared to 4% as reported by Verzoni [28] or 17.9% reported by Ishihara [29].

The spectrum of endocrinopathies reported in the literature in patients receiving ICIs is quite broad including hypophysitis, thyroiditis, and less frequently, primary adrenal insufficiency, hypogonadism, pancreatitis, hypercalcemia, and diabetes [35,36].The most frequent endocrine toxicities registered in our center in mRCC patients were thyroiditis (either as hypothyroidism or as thyrotoxicosis or both in the same patient), followed by hypophysitis and hyperglycemia. The reasons for such a high rate of endocrine toxicity are unclear. A potential explanation may be a specific search in our practice for signs and symptoms of endocrinopathies that often subtly present themselves with generic symptoms such as fatigue, nausea, and weight changes. Additionally, our patients have often undergone immunotherapy after having already experienced one or more lines of therapy with TKI, drugs that are known to often interfere with the endocrine system, especially the thyroid axis. In fact, endocrine toxicities were significantly higher in patients who had undergone multiple rounds of molecular-targeted therapies before developing endocrine toxicities while on immunotherapy. However, there are currently no studies on this topic. Further explanation could be a longer follow up period, which led to the discovery of a higher number of toxicities.

The correlation between irAEs and patient outcome has been recently described for different cancers [10,37,38]. The occurrence of irAEs has been found to be associated with favorable outcomes in melanoma [21,22,23], NSCLC [24,25,26] and, with only a few studies available, also in mRCC [27,28,29]. These studies corroborate the hypothesis of a direct link between antitumor response and auto-immune reactivity. There are two different hypothesized mechanisms that could lead to irAEs. The first one concerns preexisting self-reactive T-cells being deregulated while the second speculates a cross-reactivity between normal tissues and tumor associated antigens, which share similar targets [39,40].

However, to identify a relationship between AE and treatment response, it is important to consider AE classes individually. Although all patients experiencing irAEs of any class exhibited more favorable outcomes, only patients with thyroid dysfunction and cutaneous toxicity demonstrated significantly higher PFS.

The positive relationship between AEs of the endocrine spectrum and treatment response has been described in a few studies, both analyzed all together or individually as thyroid dysfunction. As above-mentioned, most studies have described a cohort of patients with melanoma [41] or NSCLC [42]. Less is known about endocrine irAEs as a prognostic factor in RCC [28]. Our study confirms this positive relationship with a percentage of responder patients who experienced an endocrine irAE of 64.7%. Interestingly, we noted a specific distribution of the subclasses of endocrine toxicity with respect to the response to treatment: thyroid dysfunction was confirmed to be a positive prognostic indicator while hypopituitarism, although rare, was a negative prognostic factor. However, this result should be carefully interpreted also by taking into account that endocrine toxicities showed two very different profiles between patients treated with combination therapy and monotherapy with nivolumab: thyroiditis were more represented in the group that performed only nivolumab, while all cases of hypophysitis were found in the combination therapy group. The connection that binds endocrine irAEs and efficacy is yet unknown. There are various possible hypotheses described in the literature. One of these assumes that the activation of pre-existing low-grade autoimmunity to thyroid glands could lead to thyroid irAEs and thus select patients with strong baseline immunity [43]. Another hypothesis suggests that thyroid antigens may have a common amino acid sequence with tumor epitopes. Thereby, the cross presentation of those epitopes could be associated with thyroid irAEs and this could facilitate the selection of patients who could benefit from immunotherapy [44].

The relationship between skin irAEs and treatment efficacy has also been previously described, mostly in melanoma and NSCLC [41,45]. A meta-analysis of ICI therapy in melanoma found that vitiligo was significantly associated with both longer PFS and OS [46]. In RCC, the only study that described the relation between skin irAEs and treatment response is the one by Verzoni [28]. Our data confirmed the positive association between skin irAEs and PFS with a significantly longer PFS in patients who experienced skin toxicity. Importantly, this association was confirmed in multivariable analysis, and to our knowledge, this is the first study indicating that this specific irAE represents an independent predictor of ICI efficacy in mRCC patients.

As an additional result, we described a positive correlation between the number of irAEs and patient outcome. Having ≥2 irAEs was associated with longer PFS compared with one or no irAE. This result has already been described in other retrospective real-world studies by Bouhlel [47] and Ricciuti [24], both in NSCLC. To the best of our knowledge, this study is the first to reveal this association in a mRCC cohort of patients. This finding further suggests a mechanistic association between irAEs and immunotherapy efficacy and indicates that the development of multiple immune-mediated toxicities might reflect sustained anti-tumor responses.

It could be argued that patients with longer response to treatment could develop a higher number of irAEs. In an attempt to address this potential confounding factor, we conducted a 16-week landmark analysis. The results confirmed (although the thyroid dysfunction did not reach statistical significance) that the occurrence of irAEs was significantly associated with prolonged median PFS. These findings further underline the association of early onset of irAEs and a durable clinical benefit in mRCC patients treated with immunotherapy.

The limitations in the interpretation of the study results are mainly impacted by the retrospective nature and the small sample size. Further limitations are the selection of patients, which is stricter in clinical trials than in real-life clinical practice. In addition, regarding the determination of outcome and follow up, patients enrolled in clinical trials followed regular and more frequent clinic visits as per pre-determined strict criteria, which are sometimes difficult to follow in a real-life scenario. Moreover, a precise definition and categorization of irAEs is still lacking, and the classification that we performed has not been validated or standardized.

However, despite these limitations, we were able to observe significant results that require further confirmation in prospective studies with larger cohorts.

## 5. Conclusions

Despite the small sample size, we observed that specific irAEs such as thyroid dysfunction and cutaneous reactions were associated with longer PFS and that patients that experienced more than one AE presented a better response to treatment. These results suggest that irAEs can be a surrogate marker of clinical benefit. Not every toxicity class was significantly associated with better clinical response. However, the two that showed a positive relationship (skin and thyroid), which were also the most frequent, could be very useful in a clinical context. Nevertheless, no definitive conclusions could be derived from these data considering the limited number of patients experiencing any specific class of irAEs.

Given the high percentage of endocrine, and in particular, thyroid AEs, it is essential to implement research and treatment of these particular toxicities during treatment. Since endocrine toxicities often present themselves with vague and unclear clinical pictures, close monitoring by an endocrinologist should be warranted, also given the positive correlation with the patient’s outcome and therefore the importance of continuing immunotherapy for these patients. Further prospective studies are necessary to confirm our findings and to investigate the mechanistic association between AEs and clinical response in order to improve therapy with ICIs and the management of their toxicities.

## Figures and Tables

**Table 1:** Clinical features of the study population.

Characteristics	No. of Patients (N = 43)	
	Sex	%
Male	35	81.4%
Female	8	18.6%
Age (years)		
Average	64	
Median	65	
Min–Max	45–79	
Smoker		
Yes	23	53.5%
No	20	46.5%
Performance Status at the time of diagnosis		
2	2	4.6%
1	2	4.6%
0	39	90.7%
Histology		
Clear cells	36	83.7%
Chromophobic cells	2	4.6%
Papillary	5	11.6%
Therapy line		
1	10	23.3%
2	25	58.1%
3	7	16.3%
4	1	2.3%
Outcome		
RC	1	2.3%
PR	6	13.9%
SD	11	25.6%
PD	25	58.1%

**Table 2:** Comparison between irAEs in mRCC patients treated with nivolumab or with nivolumab and ipilimumab.

irAEs	Nivolumab		Nivolumab + Ipilimumab		Total	
	n	%	n	%	n	%
Pneumonitis	3	9.1%	0	0.0%	5	6.9%
Colitis (diarrhea)	5	15.1%	0	0.0%	5	11.6%
Hepatitis	4	12.1%	3	30,0%	7	16.3%
Skin reactions	8	24.2%	1	10.0%	9	20.9%
Nephritis	0	0.0%	1	10.0%	1	2.3%
Arthralgia/myalgia	4	12.1%	0	0,0%	4	9.3%
Endocrine-related events	14	42.4%	6	60.0%	20	46.5%

**Table 3:** Univariate and multivariate analysis of progression-free survival.

Characteristics	Univariate Analysis		Multivariate Analysis	
	HR (95%CI)	*p*-Value	HR (95%CI)	*p*-Value
Age, years (≥65)	1.41 (0.66–2.99)	0.373	-	
Gender (female)	0.91 (0.34–2.39)	0.842	-	
Smoking history (current or former)	1.20 (0.57–2.54)	0.625	-	
Histopathology (clear cells)	2.22 (0.88–5.58)	0.089	2.09 (0.72–6.02)	0.173
Skin toxicity (present)	0.33 (0.11–0.95)	0.041	0.36 (0.12–1.06)	0.065
Thyroid disfunction (present)	0.36 (0.15–0.89)	0.028	0.34 (0.13–0.87)	0.025
Number of irAEs (≥2)	0.33 (0.13–0.81)	0.016	0.32 (0.13–0.79)	0.014

HR = hazard ratio; CI = confidence interval; irAEs = immune-related adverse events.

## Data Availability

Data available on request to the corresponding author.
